# Novel mitochondrial genome rearrangements including duplications and extensive heteroplasmy could underlie temperature adaptations in Antarctic notothenioid fishes

**DOI:** 10.1038/s41598-023-34237-1

**Published:** 2023-04-28

**Authors:** Bushra Fazal Minhas, Emily A. Beck, C.-H. Christina Cheng, Julian Catchen

**Affiliations:** 1 Informatics Programs, University of Illinois at Urbana-Champaign, Urbana, USA; 2 Data Science Initiative, University of Oregon, Eugene, USA; 3 Department of Evolution, Ecology, and Behavior, University of Illinois at Urbana-Champaign, Urbana, USA

**Keywords:** Molecular evolution, Genome informatics

## Abstract

Mitochondrial genomes are known for their compact size and conserved gene order, however, recent studies employing long-read sequencing technologies have revealed the presence of atypical mitogenomes in some species. In this study, we assembled and annotated the mitogenomes of five Antarctic notothenioids, including four icefishes (*Champsocephalus gunnari*, *C. esox*, *Chaenocephalus aceratus*, and *Pseudochaenichthys georgianus*) and the cold-specialized *Trematomus borchgrevinki*. Antarctic notothenioids are known to harbor some rearrangements in their mt genomes, however the extensive duplications in icefishes observed in our study have never been reported before. In the icefishes, we observed duplications of the protein coding gene *ND6*, two transfer RNAs*,* and the control region with different copy number variants present within the same individuals and with some *ND6* duplications appearing to follow the canonical Duplication-Degeneration-Complementation (DDC) model in *C. esox* and *C. gunnari*. In addition, using long-read sequencing and k-mer analysis, we were able to detect extensive heteroplasmy in *C. aceratus* and *C. esox*. We also observed a large inversion in the mitogenome of *T. borchgrevinki*, along with the presence of tandem repeats in its control region. This study is the first in using long-read sequencing to assemble and identify structural variants and heteroplasmy in notothenioid mitogenomes and signifies the importance of long-reads in resolving complex mitochondrial architectures. Identification of such wide-ranging structural variants in the mitogenomes of these fishes could provide insight into the genetic basis of the atypical icefish mitochondrial physiology and more generally may provide insights about their potential role in cold adaptation.

## Introduction

Mitochondria (mt) are specialized cytoplasmic organelles that provide substantial energy to eukaryotic cells and have enabled the evolution of eukaryotic complexity^1^. They contain their own genomes and are involved in significant biological processes like aerobic metabolism, stress response, energy balance, and oxidative phosphorylation (OXPHOS), among many others^2–5^. A typical metazoan mitogenome is very small (15–19 kilobasepairs (kbp)) having transferred many genes not needed for local metabolic control to the host organism’s nuclear genome^6^. This transfer of mtDNA is an ongoing and ubiquitous process that gives rise to noncoding sequences called NuMTs, or nuclear mtDNA^7^. With few exceptions metazoan mitogenomes possess a double-stranded, circular DNA molecule containing 13 protein coding genes, 22 transfer RNA (tRNA) genes (required for translation of proteins encoded by the mitochondrial genome^8^), two ribosomal RNA genes (*16S* and *12S*), a light strand origin of replication, and a control region (CR), which harbors transcription promoters and replication origins. Teleosts follow metazoan mitogenome architecture, but deviations have been identified involving duplications, local position changes (shuffling), and transpositions, while inversions are considered relatively rare^9–14^. Apart from gene duplications and insertion/deletion events, the majority of mt genome size variation is attributed to length differences in the CR, which might arise due to variability in the length or number of simple sequence repeats^15–17^.

Differing mitochondrial complements, called heteroplasmy, are detectable when mitogenomes possess differences in their length or nucleotide sequence and are known to arise because of somatic mutations, paternal leakage, or biparental transmission as reviewed in Breton and Stewart^18^. Somatic mitogenomic mutations are particularly prevalent as the mutation rate of mt genomes is roughly 5–10 times that of the nuclear genome^19–21^. The level of heteroplasmy can vary at different organizational levels within the individual (cells, tissues, and organs), and at a population level where different rates of heteroplasmy may be present in different individuals^22,23^. Heteroplasmy is biologically important as it results in the presence of a dynamic pool of mt genomes in an organism; it can be sustained as a result of (1) random genetic drift causing an increase in the population of a particular type of mitochondrial genome through an unbiased transmission to daughter cells^23^, (2) relaxed replication where the proportion of any mt genome variant can also increase as mitochondria are replicated and destroyed continually in non-dividing cells^23^, or (3) positive selection of a variant having a functional advantage^24^.

Antarctic notothenioid fishes—cryonotothenioids—are the principal group of teleost fishes endemic to the Southern Ocean^25^. During the last 40 million years, as the region has undergone climatic changes resulting in extreme cold environments, cryonotothenioids emerged as the dominant marine teleost taxon, having evolved various physiological and morphological adaptations^26^ the most well-studied being the antifreeze glycoproteins, which prevent the growth of ice crystals within the fish^27^. Icefishes (Channichthyidae), the most derived cryonotothenioids, are further specialized for the cold^28^, and notably are the only vertebrates that lack hemoglobin and the oxygen transport it provides^29,30^. Correspondingly, major cardiovascular changes have been identified in this group of fishes^31–33^ along with a unique mitochondrial physiology including a larger size and lower surface-to-volume ratio due to lower cristae density compared to red-blooded species^34,35^. The heart and muscle cells in icefishes contain high densities of mitochondria, and the presence of abundant organellar lipid membranes could facilitate oxygen flux into the cell and the mitochondrial matrix, given O_2_ is much more soluble in lipids than in aqueous cytoplasm^36^. The icefish mitochondria have been described as having a “unique form and function” for their special architectural features and activities^35^, even when comparing to the related, red blooded cryonotothenioids.

Despite these radical specializations for life in the cold, one species of icefish, *Champsocephalus esox*, migrated within the last two million years to warmer Patagonian waters notwithstanding the lower oxygen concentrations^33,37^. This species represents a model of *adaption following adaption*, exhibiting physiological changes for life in a temperate environment originating from an already derived icefish physiology. The mitochondria of *C. esox* are large in size like other icefishes, however, *C. esox* displays both high mitochondrial densities and inner membrane morphology different from that of other icefishes with cristae density higher and more similar to red-blooded species than other Antarctic fishes^38^. Increase in mitochondrial size or cristae density can be used to increase respiratory output^35^, thus larger mitochondria in *C. esox* with higher cristae densities might be able to generate higher respiratory output which may be related to changes involved in adaptation to warmer temperatures. Positive selection has also been observed to act on several nuclear genes related to mitochondrial function and morphology when compared to its Antarctic sister species, *Champsocephalus gunnari*^39^. These patterns of selection, combined with the observed mitochondrial phenotypes, suggest that the organelle plays a fundamental role in the adaptation to warmer, temperate environments.

Changes to the mitochondria in cryonotothenioids are not limited to icefish. The cryopelagic red-blooded bald notothen, *Trematomus borchgrevinki*, is a more basal notothenioid that forages in the platelet ice layer under surface fast ice (ice *fastened* to the coastline) of McMurdo Sound, the coldest and iciest habitats in the Southern Ocean. While stenothermal and adapted for extreme cold, the bald notothen is known to have retained some amount of plasticity in response to heat stress^40^. The mitogenome of *T. borchgrevinki* has been reported to harbor a large inversion^41^; however, the architectural details underlying this inversion, and its role (or lack thereof) in cold specialization, remain unclear.

While many specific phenotypic changes for survival in the extreme cold have been described in cryonotothenioids, the molecular mechanisms of cold adaptation are not completely understood. Moreover, the role of the mitogenome in adaptive evolution remains little explored, however there are various studies which highlight the significance of mt genomic components in compensating for changing environments, including the role of *ND6* in high altitude adaptation in Tibetan horses^42^, selection on the *ND4* and *ND5* genes to adapt to an active marine lifestyle in sea turtles^43^, and association of the *ND5* gene with cold tolerance in Chinese tiger frogs^44^.

Most of the mt genomes available today were generated by long-range PCR coupled with first-generation Sanger sequencing, or directly using second-generation short-read sequencing. Both the short lengths of second-generation reads (100–300 bp) and limitations in PCR are unable to correctly resolve the repetitive sequences of the mt CR^45,46^ or identify structural changes across the mitogenome. With the availability of third-generation, long-read sequencing the entire mt genome can be captured in a single read enabling assembly without any ambiguity. Recent studies using long-read sequencing have discovered spans of tandem repeats within the control region of mitogenomes^47,48^. Any of these mt elements that are longer than the insert length of short-reads will be unresolvable by a short-read assembler, including inversions and tandem duplications^46^.

**Figure 1:**
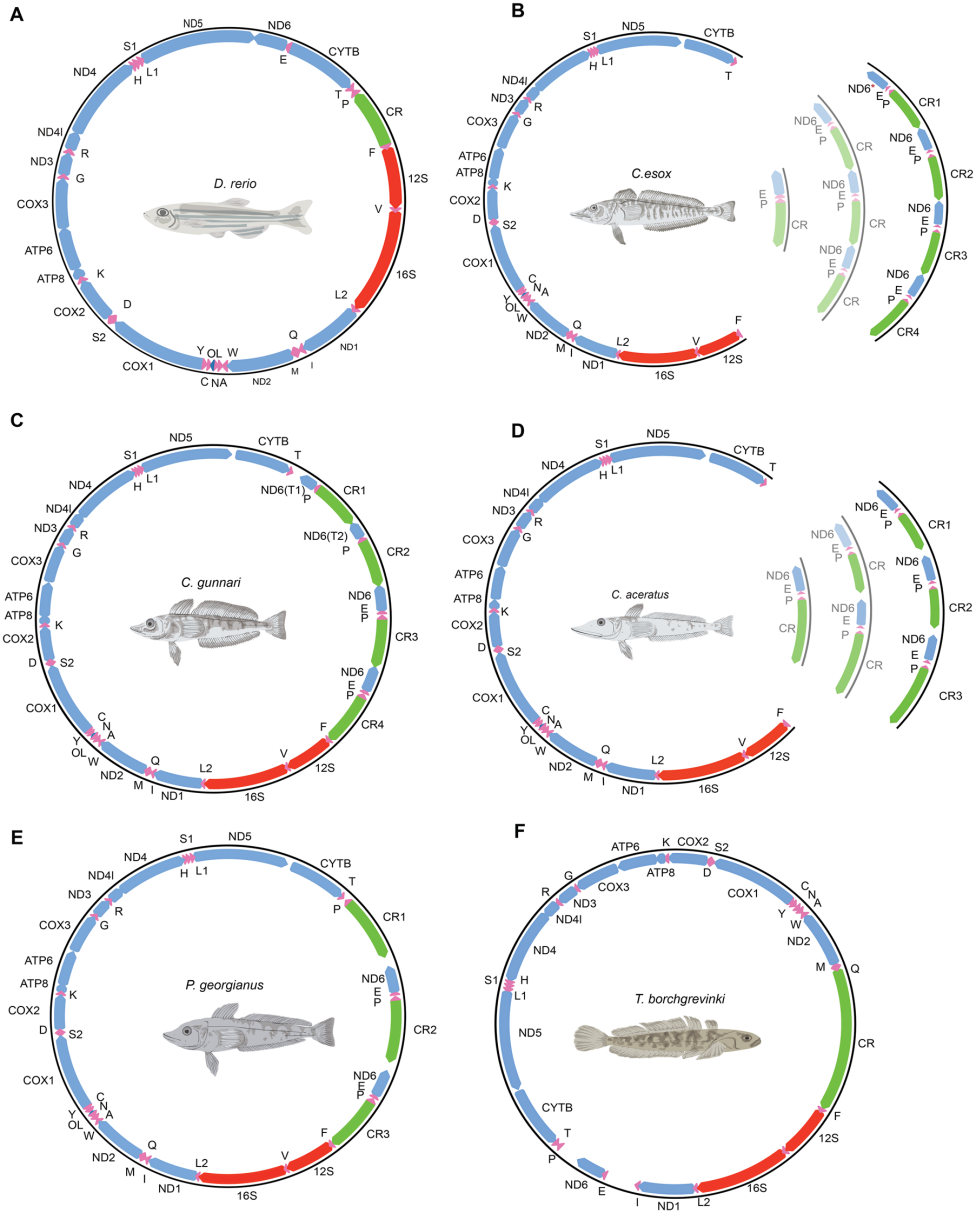
Complete annotated mitogenomes of species in this study. Protein coding genes are colored as blue, tRNAs as pink, ribosomal subunits genes as red, and control region is colored in green. The lighter colors in *C. esox* and *C. gunnari* depicts the different variants of mt genome with the darker portions showing the primary assembly for that species. (**A**) Mitogenome of *Danio rerio* showing gene order of a typical mt genome (**B**) Mitogenome of *C. esox* showing heteroplasmy where we have observed reads showing variable mitogenomes with different numbers of the tandemly duplicated region *ND6*/*trnE*/*trnP*/CR, with reads having one, three and four copies of the duplicated block. (**C**) Mitogenome of *C. gunnari* with two complete blocks of *ND6*/*trnE*/*trnP*/CR region and two blocks with *ND6-T*/*trnP*/CR where *ND6-T* depicts the truncated copy of the *ND6* gene. (**D**) Mitogenome of *C. aceratus* showing heteroplasmy with one, two, and three copies of the *ND6*/*trnE*/*trnP*/CR region. (**E**) Mitogenome of *P. georgianus* with two copies of the duplicated *ND6*/*trnE*/*trnP*/CR region, and an additional copy of just *trnP*/CR. (**F**) Mitogenome of *T. borchgrevinki* showing an expanded control region within an inversion of 6551 bp.

In this study, we analyze the assembly and architecture of the mitogenomes of five cryonotothenioids: four icefishes (*Champsocephalus gunnari*, *Champsocephalus esox*, *Chaenocephalus aceratus*, and *Pseudochaeniuchthys georgianus*) belonging to family Channichthyidae, and *Trematomus borchgrevinki* from the red-blooded subfamily Trematominae, using long-read sequencing which provides the capability to capture the majority of mt DNA within single reads (Fig. 1). This is the first report on the mitogenome of the secondarily temperate icefish *C. esox* and the first complete long-read assemblies of the other icefishes and *T. borchgrevinki.* We found the mitogenomes of cryonotothenioids have undergone extensive rearrangements, with icefishes exhibiting tandem duplications of a region containing the *ND6* gene, *trnE* and *trnP* tRNAs, and the CR (hereafter, *ND6*/*trnE*/*trnP*/CR), and *T. borchgrevinki* displaying a large inversion*,* first reported by Papetti et al.^41^, that contains a set of CR tandem repeats. We also identified potential evidence of the duplication-degeneration-complementation (DDC) model^49^ in action in *ND6* in *C. esox* and *C. gunnari* revealing the potential of these fishes as new evolutionary mutant models^50,51^ for studying OXPHOS and other mitochondrial-driven processes.

## Results

### Mitogenome assembly of *Champsocephalus esox*

**Figure 2:**
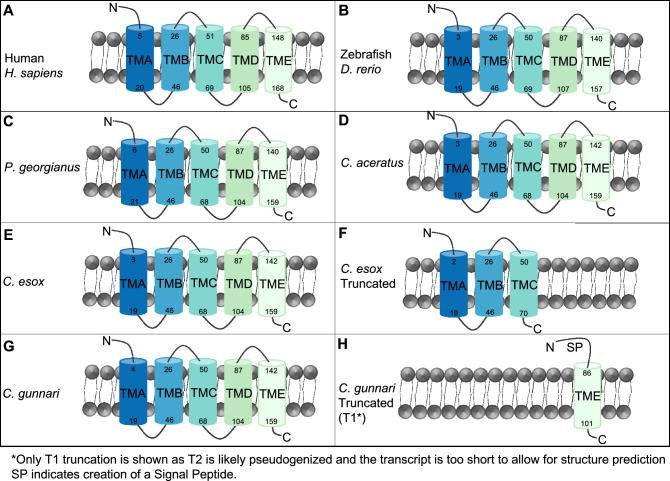
Protein structure of complete ND6 proteins contain five transmembrane proteins (TMA-TME). Protein structure of a complete ND6 protein in (**A**). Human, (**B**) Zebrafish, (**C**) *P. georgianus*, (**D**) *C. aceratus*, and (**E**) wildtype *C. esox*. (**F**) Protein structure of a truncated ND6 protein in *C. esox*, where the protein structure terminates near the midpoint of complete ND6 protein retaining wildtype structure of the N-terminal half of ND6 through TMC. (**G**) Protein structure of a complete wild type ND6 in *C. gunnari*. (**H**) Protein structure of truncated copy of ND6-T1 in *C. gunnari* where the C-terminal half with one transmembrane TME is retained along with predicted signal peptide (SP) at the N-terminal end. Only T1 truncation is shown as T2 is likely pseudogenized as the transcript is too short to allow for structure prediction.

To assemble the *C. esox* mitogenome, we mapped raw PacBio reads generated by our nuclear genome projects to the existing *C. gunnari* mt reference genome (NCBI accession NC_018340), extracted mapped reads, and assembled and annotated the result. The mt genome for *C. esox* has been reported for the first time in this study and the consensus assembly is 22,372 bp in length, containing 16 protein coding genes, 28 tRNA genes, two ribosomal subunit genes, and four CR sequences (Table 1). The increased number of genes is due to the presence of a duplicated *ND6*/*trnE*/*trnP*/CR segment in tandem four times. Out of the four control regions, the first three are about the same length as typical control regions (1004, 1001, and 1004 bp), whereas the last control region is 1097 bp in length, and the CRs did not contain any repeats. This duplication also results in four copies of the *ND6* gene along with intact *trnE* and *trnP* tRNA genes (Fig. 1B). Out of the four copies of *ND6*, one has a single base insertion, which shifts the reading frame and results in a truncated translated protein 80 amino acids in length that is different from the other three complete ND6 proteins, each 175 amino acids in length. The wildtype full length ND6 protein mirrors the structure of that seen in humans and other traditional animal models like zebrafish (Figs. 2A,B,E, S1, S2). Recent predictions using deep learning models predict a 5 transmembrane domain (TMA-E) protein which differs from the original prediction of 6 TMs^52^ (Figs. 2, S1). The truncated copy in *C. esox* terminates near the midpoint of the full wildtype protein sequence, retaining wildtype structure of the N-terminal half of ND6 through TMC (Figs. 2F, S1).

**Table 1:** Raw and canu-corrected read statistics, genome size and content of mt genomes of species in this study.

Species	Raw reads	Raw read N50	Number of corrected reads	Assembly N50 (bp)	Genome size (bp)	Protein coding genes	tRNA genes	Control regions
*C. esox*	840	10,162	61	16,096	22,372	16	28	4
*C. gunnari*	608	7706	87	8219	21,687	16	26	4
*C. aceratus*—Flye default assembly^1^	1988	14,802	441	10,118	20,561	15	26	3
*C. aceratus*—Flye assembly; two *ND6* copies^2^	304	18,154	39	18,439	19,015	14	24	2
*C. aceratus* – Flye assembly; three *ND6* copies^3^	194	19,342	35	19,558	20,637	15	26	3
*P. georgianus*	190	9182	81	9138	20,821	14	25	3
*T. borchgrevinki*	416	13,624	51	19,055	19,290	13	22	1

1. The default *C. aceratus* mt genome assembly generated from all reads.

2, 3. *C. aceratus* mt assemblies generated by reads containing 2 and 3 ND6 copies respectively (as determined by k-mer analysis).

Delving beyond the standard assembly, a k-mer analysis of raw reads enabled us to discover the presence of heteroplasmy in *C. esox*—i.e., a variable set of mitogenomes in this individual including the *ND6*/*trnE*/*trnP*/CR block tandemly duplicated up to five times. There were 59 reads spanning the *12S*—*CYTB* block (the region containing the repeated *ND6*/*trnE*/*trnP*/CR block). Two of these reads had one *ND6* copy, 11 reads contained three *ND6* copies, 33 reads had four copies, one read had five copies, and the rest of the reads spanned the other side of the mitogenome. Along with *ND6* copies they also maintained the rest of the *ND6*/*trnE*/*trnP*/CR region. While various sequence^53^ or length^54^ heteroplasmy in mt genomes have been reported, as far as we know, *C. esox* mt heteroplasmy is the most extensive case, with co-occurrence of different variants of mt genomes, each carrying variable numbers of genes (in variable numbers of duplicated blocks).

### Mitogenome assembly of *Champsocephalus gunnari*

The mitogenome for *C. gunnari* was assembled by collecting long, raw reads that mapped to the existing *C. gunnari* reference genome (NCBI accession NC_018340 and assembling and annotating them de novo. The mt genome of *C. gunnari* is 21,687 bp in length, 2.8 kbp longer than the 18,863 bp reference. The genome consists of 16 protein coding genes, 26 tRNA genes, two ribosomal subunit genes, and four CRs (Table 1). The greater number of protein coding genes, tRNA genes, and CRs are due to a duplicated region containing *ND6*/*trnE*/*trnP*/CR four times in tandem (Fig. 1C), similar to *C. esox* but with important differences. Three of the four CRs have lengths of 1002, 1003, and 1004 bp, whereas the last CR is a larger sequence of 1103 bp. Like *C. esox*, the CRs also did not contain any tandem repeats. In *C. gunnari*, however, we observed an alteration of the start site for *COX1* from an ATG to a GTG start codon and, more importantly, observed two complete *ND6*/*trnE*/*trnP*/CR duplicated blocks along with two additional blocks again containing truncated copies of the *ND6* gene and a loss of *trnE* (Fig. 1C).

In both truncated copies of *C. gunnari ND6* there is an insertion in the same region as *C. esox* leading to a frameshift mutation, but *C. gunnari* also contains additional mutations upstream of the insertion resulting in alternative start sites, one in each truncated copy. Both truncated copies contain a transversion converting ATC to ATG resulting in a new open reading frame and additional unique insertions. In one truncated copy (T1), there is an insertion of three Gs leading to a partially conserved C-terminal protein sequence of ND6 including TME. In the second truncated copy (T2), there is an insertion of four Gs. In this copy, the insertion of four nucleotides results in a frameshift leading to a possible transcript 69 bp in length (Figs. 2H, S2).

Despite a highly derived mitogenome architecture, we did not find any heteroplasmy in the *C. gunnari* mitogenome.

### Mitogenome assembly of *Chaenocephalus aceratus*

**Figure 3:**
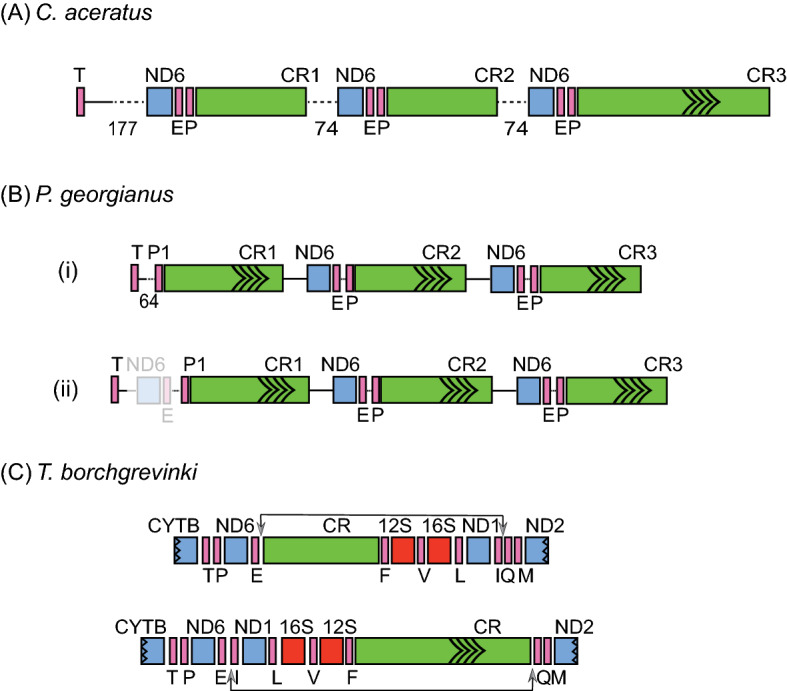
Duplication topology. (**A**) Region showing the tandem duplicated block for *C. aceratus* with repeats in CR3. (**B**) (i) Region containing the tandem duplicated block for *P. georgianus* and all the control regions with repeats. (ii) The light colored region between T and P shows the potential copy of *ND6* and E that might have been lost. The dotted region between E and P1 matches the region between E and P in retained copies thus showing the presence of third copy of the *ND6*/*trnE*/*trnP*/CR block which is partially lost. (**C**) Comparison of the reference (top) versus our mt genome of *T. borchgrevinki* (bottom). The arrows on top show the position of the region which is inverted in our assembled genome. The arrows in the CR are the tandem repeats.

The *C. aceratus* mitogenome was assembled by mapping raw, long reads from the *C. aceratus* nuclear genome library (BioProject PRJNA420419) to the reference *C. aceratus* assembly (NCBI accession NC_015654.1). The mapped reads were then extracted, assembled and annotated. The length of the primary *C. aceratus* mt genome assembly presented in this study is 20,561 bp, 3.2 kbp longer than the NCBI reference (17,311 bp). It consists of 15 protein coding genes, 26 tRNA genes, two ribosomal subunit genes, and three CRs (Table 1). We observed three copies of the *ND6*/*trnE*/*trnP*/CR region present in tandem, in contrast to a single copy found in the reference genome. It appears the region underwent tandem duplication starting from the intergenic space between *trnT* and *ND6* and ending at the CR (Fig. 3A). We also observed a tandem duplication in one of the CRs, which is longer (1306 bp) compared to the other two CRs which are both 847 bp (Fig. 1D; Table S1).

We observed heteroplasmy in *C. aceratus* using k-mer analysis, where we observed six reads having one *ND6* copy, 304 reads with two *ND6* copies, 193 reads with three *ND6* copies, and one read with four *ND6* copies. Each *ND6* copy occurs in a *ND6*/*trnE*/*trnP*/CR block, thus the variants with multiple *ND6* copies contain duplicated *ND6*/*trnE*/*trnP*/CR blocks. Two of the four element layouts were captured in enough reads to perform standard assemblies. These two sets of reads produced two different mt genomes, one mt genome assembly with length 19,015 bp and contained two copies of the *ND6*/*trnE*/*trnP*/CR block, while the other mt genome was 20,637 bp in length and recapitulated three copies of the *ND6*/*trnE*/*trnP*/CR region (Fig. 1D; Table 1).

### Mitogenome assembly of *Pseudochaenichthys georgianus*

We assembled the *P. georgianus* mitogenome by mapping the raw, long reads from the nuclear genome library (BioProject PRJEB19273) to the reference *P. georgianus* assembly (NCBI accession NC_057673.1). The mapped reads were then extracted, assembled, and annotated. The mt genome of *P. georgianus* is 20,821 bp in length, 3.5 kbp larger than the reference genome, which is 17,310 bp. The mitogenome contains 14 protein coding genes, 25 tRNA genes, two ribosomal subunit genes, and three CRs (Fig. 1E; Table 1). We observed two copies of the *ND6*/*trnE*/*trnP*/CR region present in tandem. We also observed an additional *trnP* and CR which seems to be the remnant of a third copy of *the ND6*/*trnE*/*trnP*/CR block. In other icefishes, *trnT* is followed by *ND6*, while in *P. georgianus,* it is followed by *trnP* instead. There is also an intergenic space between *trnT* and *ND6* that is of variable length (304 bp in *C. esox,* 197 bp in *C. gunnari*, and 177 bp in *C. aceratus*). In *P. georgianus,* the space between *trnT* and *trnP* is only 64 bp. When analyzed, a part of this region (from 38 to 64 bp) was identical to the intergenic region between *trnE* and *trnP*. The reduced length and the presence of the intergenic region between *trnE* and *trnP* might indicate the prior presence of a third copy of *ND6*/*trnE*/*trnP*/CR block, which may have been lost partially, removing most of the intergenic space between *trnT* and *ND6*, the *ND6* gene, and *trnE* while retaining the intergenic space between *trnE*, *trnP*, and the CR (Fig. 3B). The three CRs (two from the intact blocks and a third from one of the two remnant blocks) are larger in size compared to the typical CRs of fish mt genomes and are of variable lengths: 1439, 1377, and 1157 bp for CR1, CR2, and CR3 respectively. This variability in length is attributed to the presence of tandem repeats, where CR1 has a tandem repeat of 53 bp repeated 8.8 times, CR2 has repeat of 53 bp that is repeated 7.8 times, and CR3 has a repeat of 63 bp that is repeated 2.1 times (Table S2). This is in stark contrast to *C. esox* and *C. gunnari* which do not contain tandem repeats.

Despite a highly derived mitogenome architecture, we did not observe any heteroplasmy in *P. georgianus.*

### Mitogenome assembly of *Trematomus borchgrevinki*

We assembled the mt genome for *T. borchgrevinki* by mapping the raw, long reads generated in our nuclear genome project to the reference *T. borchgrevinki* mt genome (NCBI Genbank accession KU951144.1). The mapped reads were then extracted, assembled and annotated de novo. The architecture of the *T. borchgrevinki* mitogenome was distinct from the icefishes as well as the typical, teleost architecture. We assembled the longest mitogenome to date for *T. borchgrevinki* and within it we observed an inversion 6551 bp in length along with tandem repeats within the control region. The mt genome of *T. borchgrevinki*, first reported by Liu et al.^55^ (NCBI accession KU951144.1; 17,299 bp), was assembled using Sanger sequencing and did not report any rearrangements. Later, Papetti et al.^41^ (NCBI accession MT232659.1; 18,325 bp) generated an additional mt genome of *T. borchgrevinki* using long-range PCR coupled with short-read sequencing and reported an inversion of at least 5300 bp. Recently Patel et al.^56^ also generated a *T. borchgrevinki* mitogenome (NCBI accession MZ779011; 18,981 bp) using Illumina TruSeq synthetic reads (short-reads combined with a scaffolding technique) and found the same inversion, along with the presence of some intergenic spacer sequences. Using our PacBio long-reads we assembled a mitogenome of length 19,290 bp, which is 309 bp longer than the mt genome assembled by Patel et al.^56^, and the gene order and content are consistent with their findings. None of the previous assemblies reported the presence of tandem repeats in the mt genome of *T. borchgrevinki*.

Like the canonical vertebrate mt genomes, the *T. borchgrevinki* mitogenome contains 13 protein coding genes, 22 tRNA genes, along with two ribosomal subunit genes, and one CR (Fig. 1F; Table 1). Apart from *COX1* (which starts with a GTG codon), all protein coding genes use the ATG starting codon. The *CYTB*, *ND4*, and *COX2* genes do not have complete stop codons, which is a common observation in vertebrate mitogenomes, as they are known to be created via post-transcriptional polyadenylation^57^. The CR for this genome was substantially longer compared to its reference (i.e., 2651 bp versus 1212 bp) and is also substantially longer than the usual CR in teleost fishes. For example, the CR for zebrafish is only 950 bp^58^. The size of the CR was verified by examining the region between *12S* rRNA and *ND2* (which spans the CR) in raw PacBio reads. We found the length was consistent with our assembled CR and we did not observe any length heteroplasmy. The CR expansion is due to the presence of a high number of repeats which spanned 1400 bp of the total length of the control region (2651 bp). There were two sets of repeats, one was 291 bp long (spanning 157–448 bp in the CR) and contained three copies of a secondary tandem repeat of 97 bp. The second repeat block spans a region from 1263 to 2381 bp in the CR and contains additional nested repeats of variable length (Fig. 3C; Table S3).

### Large Inversion in *T. borchgrevinki*

Similar to what was reported by Papetti et al.^41^, we also observed a large inversion in the mitogenome, though we found the inversion to be 1.2 kbp longer (6551 bp in our data compared to 5300 bp). The difference in length is attributed to the expanded control region containing tandem repeats. (Fig. 1F) which is also the region that harbors the machinery for mitogenome replication. We manually assigned the putative OriL at the expected location (between the *trnN* and *trnC* tRNA genes; see Methods); it was considerably shorter in length (25 bp) and did not form its usual hairpin structure.

### Evolution of duplicated genes in icefishes

**Figure 4:**
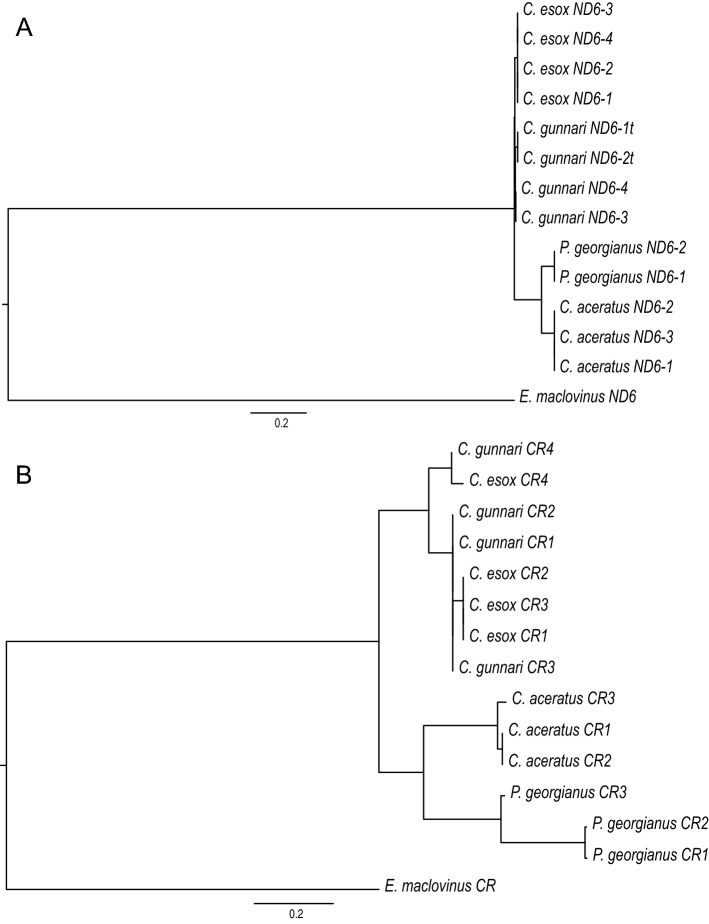
Phylogenetic relationships among mt elements as observed using PhyML (BIC). (**A**) Phylogenetic tree of *ND6* genes of four icefishes with *E. maclovinus* as outgroup. (**B**) Phylogenetic tree of mt control region of four icefishes with *E. maclovinus* as the outgroup.

To understand the evolutionary origin of the duplicated *ND6* copies and CRs, we performed a phylogenetic analysis of these genes and CRs with *Eleginops maclovinus* as an outgroup. We observed that the *ND6* copies are more closely related within a species compared to their respective orthologs. Similarly, for CRs, the paralogs group together for each species showing that paralogs are more closely related than orthologs across species, except for the last CR of *C. gunnari* and *C. esox* which are longer than the first three CRs in both species and thus group together (Fig. 4).

## Discussion

### Presence of heteroplasmy could provide a reservoir for selection

Through long-read sequencing coupled with k-mer analysis, we demonstrated mt genome heteroplasmy in icefishes for the first time. The presence of more than one kind of mitogenome was originally considered to be a rare phenomenon and often associated with aging and disease^59^ (reviewed in Elorza and Soffia^60^) but recent studies show that it may be more prevalent than originally thought^61,62^. It is quite challenging to study the effects of heteroplasmy because it is difficult to associate phenotypic effects with different genetic copies present at low frequencies. The challenge is particularly acute since a mitogenome might not render any phenotypic effects unless it reaches a certain prevalence threshold as reviewed in Rossignol et al.^63^ The discovery of more than one mitogenomic variant in two of the four icefishes we examined demonstrates heteroplasmy, but our current approach does not tell us at what organizational level the heteroplasmy is occurring: it could occur within a specific tissue or organ, but it may also be present at a certain life stage across tissues, or the variants may be fixed within an individual but segregating across the population. A simpler reason we did not identify heteroplasmy in the remaining icefishes may simply be due to insufficient depth of mt sequencing, or the particular tissue or cell type that was used for sequencing. Mitochondrial heteroplasmy has been observed previously in humans^64^, Tuatara^53^, bat^65^ and other species (reviewed in Barr et al.^66^) but it has been mostly limited to minor variations in length or nucleotide sequence.

For this study, we used mt reads that were sequenced incidentally with the nuclear DNA for the tissues chosen—white muscle for *C. gunnari*, *C. aceratus*, and* T. borchgrevinki*, hepatocytes (liver) for *C. esox*, and spleen for* P. georgianus*. These tissue types are quite different, with liver being the most metabolically active, followed by spleen. However, heteroplasmy was detected in *C. esox* and *C. aceratus*, demonstrating that it is occurring in disparate tissue types across at least two of the icefishes. More targeted, and tissue-specific sequencing may provide further details as to the frequency of heteroplasmy.

### Gene duplications may lead to subfunctionalization in OXPHOS complex I

Vertebrates have evolved a very compact mitogenome, with very few intergenic sequences, and small numbers of rRNA and tRNA genes^67,68^. The conservation of small-sized vertebrate mitogenomes implies that mt gene duplications should be a rare phenomenon. However, as more mitogenomes are constructed from long reads, evidence is accumulating that mt gene duplications are prevalent^69,70^. The formation of multiple copies of the *ND6*/*trnE*/*trnP*/CR region in four icefishes and heteroplasmy in at least two species of icefish imply that these additional copies may have functional relevance. Duplications within the nuclear genome are major contributors to adaptive evolution by generating genes which can acquire novel functions^71^. These duplicated copies are subjected to the same evolutionary forces and tend to diverge over time. In the vertebrate nuclear genome, duplicated genes are fated to pseudogenization, subfunctionalization, or neofunctionalization in rare instances, however this process is not as well understood for mt genes. Here we report possible subfunctionalization or subfunction partitioning in *ND6* in *C. esox* and *C. gunnari.*

In *C*. *esox* we find evidence for possible subfunction partitioning*.* Specifically*,* we identified a truncated copy of *ND6* resulting from a frameshift mutation that retains the N-terminal half of the ND6 protein. Secondary structure and TM predictions demonstrate that this is enough of the protein to maintain a wildtype N-terminal protein structure (Figs. 2F, S1, S2). This is genetically important because the structure of the ND6 protein is highly conserved. The general structure—conserved from icefishes, to zebrafish, to humans—includes five predicted Transmembrane domains (TMA-E). In *C. esox*, the truncation results in a wildtype structure through TMC. This is functionally important because ND6 plays several important roles in the assembly and function of the OXPHOS Complex I. ND6 functions at a very specific hinge point of Complex I helping to regulate the physical relationship between the peripheral and membrane arms. The physical relationship of these arms determines if Complex I is in a closed (active) or open (deactive) state. The transition from closed to open is partially facilitated by TMC of ND6 with the deactive state being defined by the relocation of TMD which arrests the enzyme in the deactive conformation^72^. Without TMD it is possible the *C. esox* truncated *ND6* copy results in a persistently closed and active state of Complex I. Interestingly, mutations almost identical to the one in *C. esox* have been functionally tested in mouse cell lines. In this case a truncation to amino acid position 79 caused by a frameshift (contrasted to position 80 in *C. esox*) resulted in decreased function of Complex I^73^. It is therefore possible that the truncated version of ND6 in *C. esox* could functionally impact OXPHOS outputs in either direction. More work is needed to characterize if this protein is being made and, if so, how it impacts Complex I. The persistence of multiple, full copies alongside the N-terminal truncated copy of ND6 in *C. esox* could therefore serve as an evolutionary mutant model (EMM) to study Complex I activity and help understand the relationships between membrane and peripheral arm function as well as complex assembly. Complex I may have some significance in response to selection, as there are various studies establishing the link of complex I genes in adaptation to various environmental stresses^42–44^. Recent studies have also shown that complex I activity decreases at high temperatures and they are a major source for the synthesis of ATP in fishes in contrast to complex II^74^. Furthermore, it has been hypothesized that transitions to an open (deactive) state occur at elevated temperatures^72^. The maintenance of an ND6 protein copy incapable of switching to an open (deactive) state could therefore have been part of a *C. esox* adaptation to warmer water.

We also observed two truncations of the *ND6* gene in *C. gunnari*, each accompanied by their own set of mutations leading to alternate reading frames and different levels of total gene truncation. In both truncated copies there is a new start site followed by different insertions. In one copy (T1) there is an insertion of a triplet of G nucleotides that results in a normal protein sequence containing only the C-terminal half of ND6 (in contrast to the N-terminal half preserved in *C. esox*)*.* In the second copy (T2) there is a frameshift leading to only a small transcript or protein being possibly produced. It is more likely that this is evidence of pseudogenization and the RNA is degraded and never translated if it is transcribed at all. (Figs. 2H, S1, S2). Importantly, the T1 truncation retains TME and also results in a predicted signal peptide at the N-terminal end which could be used for specifying different cellular placement of ND6^75^. This set of mt protein truncations in *C. gunnari* seems genuinely novel, with the physiological implications unknown, providing a unique model to better understand how and if changes to Complex I, and its constituent proteins, are tied to changes in environment. Still more work is needed to confirm if the T1 and T2 truncated copies of ND6 are translated or functional.

The *ND6* gene is an important component of Complex I of the mt electron transport chain and OXPHOS pathway, and changes in its structure might lead to disease^73,76^. Mutations in the *ND6* gene were found to be responsible for the generation of hypoxia sensitive tumor cells in a human tumor study, and the function of ND6 within Complex I was associated with this hypoxia response^77^. Another study^78^ has identified *ND6* as a hub of initiation of replication in chickens. Given the significance of *ND6* in mitochondrial function, the presence of duplicated copies of *ND6* in icefishes might indicate a potential role in surviving extreme temperatures.

### Concerted evolution

Because of the tight functional connections between protein coding genes, tRNAs, and the CR, we might expect some mt regions to evolve together by concerted evolution. Phylogenetic analysis of the duplicated *ND6* genes and CRs shows that the paralogous copies are more similar and tend to group together compared to their respective orthologs, thus suggesting concerted or parallel evolution. It also implies that duplication may have occurred independently in each species. This may imply that we fortuitously encountered these duplications before they were fully pseudogenized, which would make them an interesting case of an EMM for OXPHOS functionality. Alternatively, the gene duplications may have occurred in the common ancestor of these icefishes. It is interesting that we have seen the same block of the mitogenome duplicated to different degrees in four species of icefish—representing two separate families, which could indicate either common ancestry of this duplication, or strong selection for the functionality underlying the duplicated block.

A third possibility is that these duplications are not segregating at the population level and are therefore recent, specific, and possibly occurring frequently in the sequenced individual. Somatic copy number variations may be selectively unimportant and very common—we just have not seen them in sequencing data until now. A key question our work raises: do copy number variants reoccur in each generation, are they tissue-specific, or are they present in the germ line—eggs or sperm. Is evolution favoring distinct sets of mt for different tissues or developmental stages within an individual or is the mtDNA evolving at a species level in response to environmental changes or are these variants simply the result of the drift of somatic mutations over time and therefore frequent and unremarkable. The *ND6* and CR duplication have been observed in the mt genomes of various birds, including cranes^79^, parrots^80^, ardeid birds^81^, and seabirds^82^. Though some possible advantages for the presence of multiple CRs have been proposed in these studies, speculation has not included any functional implications for multiple *ND6* copies. In each of these cases the *ND6*/CR duplications have been attributed to species-level, concerted evolution, which implies the presence of some form of recombination in mitogenomes, and mechanisms that might involve gene conversion events or gene turn over^83^.

Beyond genes involved in the formation of Complex I, the presence of multiple CRs might infer a functional need to alter mt replication and transcription. Such changes could provide an advantage of increased mt genome copy number per organelle, an increased rate of replication^84^, or may play a role to increase metabolic rates in response to environmental stress^85^. Various studies have linked the presence of duplicated CRs with efficient replication mechanisms^86,87^. In birds, the presence of multiple CRs is associated with longevity^85^ and confers efficient mt functionality and increased energy production required for active flight^80^. In human cell lines that have been modified to contain mitogenomes with duplicated control regions, they were able to outcompete cell lines without the duplications^88^. It has been hypothesized that multiple CRs might play a role in the survival in extreme conditions by adapting to higher energy/metabolism needs as a result of improved replication and transcription^47^.

### Tandem repeats in *T. borchgrevinki* CR may provide a selective advantage

While the icefishes show evidence for the duplication of whole CRs, *T. borchgrevinki* contained a single, canonical CR, but its length was expanded by extensive tandem repeats (Table S3). The use of long-read sequencing enabled us to assemble the repeat repertoire in mt genomes with increased confidence^48^. In the absence of any selective advantage, small mt genomes would be favored for fast replication, but the presence of larger mt genomes formed from an expanded control region (with repeats) might infer a selective advantage. Although the biological significance of these repeats is unclear, they are known to harbor elements that regulate replication/transcription. The expanded CR may be involved in better replication efficacy, improved transmission, or enhanced energy maintenance mechanisms, however more comparative work needs to be completed to understand if and to what extent these repeats are segregating in the larger population of *T. borchgrevinki* and how well these CR tandem repeats are preserved across the notothenioid clade.

### The *T. borchgrevinki* mitogenome is dominated by a large inversion

As first reported by Papetti et al.^41^, we also observed a large, inverted segment in the mt genome of *T. borchgrevinki* containing the CR along with *trnF*, *12S*, *trnV*, *16S*, *trnL*, *ND1* and *trnI*. Mt genomes are known to have a different base composition (either rich in G/T or alternatively A/C nucleotides) in their light and heavy strands, which results from an asymmetrical mutation process^89^. While the implication of such inversion in mitochondrial functioning cannot be confirmed, an intragenic inversion in the *ND1* gene is known to be linked to mitochondrial myopathy^90^. The inversion of the CR is however associated with changes in nucleotide composition of protein coding genes and is known to cause some level of reversal in compositional bias^41,91^. One explanation for the changes in compositional bias could be a reversal of the replication processes due to the physical inversion (and reverse complementation) of the CR. We also noticed alterations in the number of genes on the heavy and light strands compared to the canonical vertebrate mt genome. Usually, all the genes in the mt genome are on the heavy strand with only *ND6* and a few other transfer RNAs encoded on the light strand. But here the inversion resulted in the transfer of the genes in the inverted block to the strand containing *ND6.*

### Long-read sequencing has enabled the detection of rearrangements and structural variants

Most mitogenomes available today have been sequenced using short-read sequencing technologies which are unable to resolve complex regions containing duplications or extensive repeats^46,47^. Our use of long-read sequencing has enabled us to assemble long tandem repeats and duplicated regions of the mitogenome and provided a platform to explore heteroplasmy. We were able to generate reads that spanned the full length of the mitochondria and in enough volume in some species to detect multiple heteroplasmic genomes in a single individual. In *C. esox* and *C. aceratus* our mt reads had an N50 length of 10,162 and 14,802 bp respectively, and the libraries in *C. aceratus* allowed us to assemble two independent genomes containing different numbers of tandemly duplicated *ND6*/*trnE*/*trnP*/CR regions. Our evidence for heteroplasmy may not reflect the full extent of the heteroplasmic conditions, as our sequencing libraries were designed to assemble nuclear genomes, and incidentally yielded only a small fraction of mtDNA reads. Long-read libraries enriched for mtDNA would be a robust approach to characterize the full magnitude of mt heteroplasmy. *T. borchgrevinki* demonstrates the utility of long-read mt libraries as the previously available short-read assemblies did not initially show the inversion, and later did not uncover the set of extensive tandem repeats in the CR^41,55,56^. Instances of mt CRs with tandem repetitive elements have been observed in various animal mt genomes^48^, but this still might be underreported because of a lack of long-read-assemblies.

### K-mer analysis enabled detection of heteroplasmy

Our k-mer analysis enabled us to visualize the variable number of *ND6* copies in the tandemly duplicated *ND6*/*trnE*/*trnP*/CR region that we would have been unable to explore if we merely relied on the consensus sequence output by standard genome assemblers. For instance, for the *C. aceratus* genome, Flye generated a mitogenome assembly containing three copies of the *ND6* gene, even though a substantial number of reads (304) contained only two copies *ND6*. As Flye works by first creating a repeat graph (by collapsing repeat sequences) and then fills in the unique segments between repeat regions^92^, it collapsed *ND6* and the CR. The k-mer analysis we performed could distinguish reads with two and three copies and led to the identification of heteroplasmy.

## Conclusion

Mitochondria are known to have a role in the adaptation to changing environmental conditions because of their significance in important life processes^2,93^. The power of mt genome adaptations to respond to extreme environments has been documented in cases of adaptation to high altitudes in Tibetan humans, horses, sheep and antelope, plateau pika, in response to altitude and cold temperatures in Chinese snub-nosed monkeys (reviewed in Luo et al.^94^), cold stress in insects^95^ and in other environmental stresses like temperature, hypoxia, and toxins in other animals^93^. Our application of long-read sequencing technology to mitochondria has highlighted a more complex genomic landscape in the mitochondria of Antarctic notothenioid fishes revealing potentially tissue- or organ-specific mitogenomes; future work must detail any functional changes resulting from the underlying heteroplasmy and determine if these genomes are reproduced somatically every generation or are part of the notothenioid germ line.

## Materials and methods

### Mitochondrial sequence reads sources and sample preparation

The collection, handling, and tissue sampling of *C. gunnari*, *C. esox*, and* T. borchgrevinki* complied with University of Illinois, Urbana-Champaign IACUC approved Animal Use Protocols 07053 and 17148. All methods reported in this study are in accordance with ARRIVE guidelines. We obtained mitochondrial sequences for the five selected cryonotothenioids from the whole genome raw read datasets of the respective species, summarized in Table 2 and detailed below.

**Table 2:** Sample information, sequencing technology and N50 raw read length for species sequenced in this study.

Species	Sample location	Sample tissue	Technology	N50
*C. gunnari* (male)*	West Antarctic Peninsula	White muscle	PacBio Sequel II	29.8 kbp
*C. esox* (male)*	Puerto Natales (Patagonia water)	Isolated hepatocytes	PacBio Sequel II	24.3 kbp
*C. aceratus* (female)	King George Island, Antarctica	Muscle tissue	PacBio Sequel	22.2 kbp
*P. georgianus* (female)	West Antarctic Peninsula	Spleen	PacBio Sequel	9.8 kbp
*T. borchgrevinki* (female)*	McMurdo Sound, Antarctica	White muscle	PacBio Sequel II	33.5 kbp

The * refers to the mt genomes assembled by the PacBio reads generated in our study. For *C. aceratus*^96^ and *P. georgianus*^97^, we downloaded the available PacBio reads and assembled the mt genomes.

For *C. gunnari* and *C. esox*, whole genome raw reads were generated by de novo sequencing for our companion genome projects. A single male specimen of *C. gunnari* caught from Gerlache Strait, West Antarctic Peninsula was sequenced using high molecular weight (HMW) DNA extracted from frozen white muscle. For *C. esox*, a single male specimen obtained from the Patagonia water near Puerto Natales, Chile was sequenced using HMW DNA derived from isolated hepatocytes. Methods of DNA preparation, Pacific Biosciences continuous long read (PacBio CLR) library construction, and sequencing on PacBio Sequel II instruments (2 SMRT cells each) are detailed in Rivera-Colón et al.^39^. Briefly, sequencing yielded 10.7 million raw reads for *C. gunnari* with a mean and N50 read length of 29.7 kbp and 29.8 respectively. For *C. esox*, sequencing yielded 12.1 million raw reads with a mean and N50 read length of 13.1 kbp and 24.3 kbp N50 respectively.

For *C. aceratus*, we obtained the sequenced PacBio Sequel data available as BioProject PRJNA420419, from NCBI accessions SRR6942631 and SRR6942632. The data were derived from sequencing genomic DNA isolated from muscle tissue of a single female fish collected from Marian Cove, King George Island, Antarctica. The prepared genomic libraries were sequenced on PacBio Sequel System using P6-C4 sequencing chemistry^96^. The BioProject provided 6.5 million raw reads, with a 13.6 kbp mean and 22.2 kbp N50 read length.

For *P. georgianus*, whole genome PacBio raw reads were available from NCBI under BioProject PRJEB19273, and accessions ERR3197127 and ERR3197122. The data were derived from sequencing DNA isolated from frozen spleen of a single female collected from the coast of Low Island, West Antarctic Peninsula. A PacBio CLR library was prepared using PacBio SMRTbell Template Prep Kit 1.0, and sequenced on a PacBio Sequel^97^. The BioProject provided 7.4 million raw reads, with a 7.1 kbp mean and 9.8 kbp N50 read length.

For *T. borchgrevinki*, de novo sequencing was carried out for this study using a single female specimen caught from McMurdo Sound, Antarctica (78°S). HMW DNA was extracted from liquid nitrogen frozen white muscle using Nanobind Tissue Big DNA kit (Circulomics), lightly sheared for a ~ 75 kbp target, and used for PacBio CLR library construction. The library was selected for inserts ≥ 25 kbp using the Blue Pippin (Sage Science) and sequenced on one SMRT cell on PacBio Sequel II system using Sequel chemistry v.2 with 30 h of data capture. The sequencing yielded 7.7 million raw reads, with 23.7 kbp mean and a 33.5 kbp N50 read length. Library construction and sequencing were carried out at the University of Oregon Genomics & Cell Characterization Core Facility (GC3F).

### Genome assembly and annotation

For all five notothenioids, raw reads were mapped against available mt reference genomes using minimap2^98^. *C. gunnari* and *C. esox* were both mapped against *C. gunnari* (NCBI accession NC_018340)^99^. The *C. aceratus* and *P. georgianus* raw reads were mapped to NCBI accessions NC_015654.1^100^ and NC_057673.1^41^, respectively, *T. borchgrevinki* was mapped against NCBI accession KU951144.1^55^. For *C. esox*, *C. aceratus, and P. georgianus*, reads with a mt matching block of at least 5000 bp were extracted using seqtk (https://github.com/lh3/seqtk) in order to assemble the mitogenome. For *C. gunnari* and *T. borchgrevinki*, reads with a matching block of at least 3000 bp were extracted the same way. By only selecting long reads mapping to a substantial portion of the reference mt genome (3000 bp for *C. gunnari* and *T. borchgrevinki*, and 5000 bp for *C. esox*, *C. aceratus, and P. georgianus*), we made sure to avoid NuMT contamination. Each set of raw reads (Table 1) were corrected using the corrections module of the Canu 1.8 assembler which improves the base accuracy in the reads^101^, and the corrected reads were then assembled de novo using the Flye assembler (v2.7)^92^. The genomes were then annotated using Mitos2^102^, and tRNAscan-SE 2.0^103^. To avoid any inconsistencies and ambiguities, the annotations were manually checked using NCBI blastn^104^. We were unable to annotate the origin of replication for light strand (OriL) for *T. borchgrevinki*. Thus we searched for it around its canonical location, and by using RNAstructure^105^ we identified a region with a hairpin structure, which we assigned as the putative OriL. The non-coding regions were explored for putative repeats using Tandem Repeat Finder (TRF)^106^.

### K-mer analysis

The icefish mt genome assemblies indicated tandem *ND6* gene duplications. To confirm the assembly results and analyze the genic architecture of this region of the mitogenome we used k-mer analysis, which would allow us to search for *ND6* genes directly within the raw reads while allowing for sequencing errors. For each icefish we used the annotated *ND6* gene as query and the raw reads as subjects of the search; we k-merized both and searched for blocks of matching k-mers. To find the number of *ND6* copies and avoid random k-mer matches, we used the number of nucleotides between consecutive blocks of k-mer matches as a threshold for defining the start and end of putative *ND6* genes. For the k-mer size 19, we set the threshold of 800 nucleotides, that is if two matching k-mers are more than 800 nucleotides apart, they are considered parts of different *ND6* genes.

The variable length of raw, PacBio long-reads was problematic in visualizing the number of copies of *ND6* genes per read. To avoid the problem of variable read lengths, which may encompass different subsets of mt genes, we extracted reads containing both the *12S* and *CYTB* gene boundaries enclosing the *ND6*/*trnE*/*trnP*/CR tandem duplicated block, and then calculated the number of gene copies present in reads that spanned this full region. In the case of *C. aceratus* and *C. esox* our k-mer analysis indicated the presence of different numbers of *ND6* genes in different reads. For *C. aceratus,* apart from the generation of a primary assembly as discussed above, we separated the raw reads containing two or three putative *ND6* copies (Table 1) and assembled mt genomes for them independently using the same methods as explained above. For *C. esox,* however we did not have enough reads for each mt genome variant needed by the assembler to assemble the complete mitogenomes separately.

### Phylogenetic analysis

The *ND6* sequences and control regions from all the icefishes were aligned with those of the basal, non-Antarctic notothenioid *E. maclovinus* (NC_033386.1) as an outgroup (Cheng, et al. in prep for *E. maclovinus assembly*). We used Geneious 2022.1.1 (https://www.geneious.com) and aligned the sequences using MUSCLE^107^ with default parameters. The alignment was then used to make a phylogenetic tree using PhyML^108^ using default settings with automatic model selection (BIC) and fast likelihood-based method branching support, the tree was visualized and re-rooted in Figtree (http://tree.bio.ed.ac.uk/software/figtree/).

### Protein structure predictions

Protein sequences of ND6 from human (*Homo sapien*) and zebrafish (*Danio rerio*) were obtained from Ensembl version 107^109^ and compared to ND6 sequences from *C. aceratus*, *P. georgianus*, *C. esox*, and *C*. *gunnari* in this study, which contain *ND6* gene duplications. Full length ND6 amino acid sequences for each species and truncated ND6 sequences from *C. esox* and *C. gunnari* were run through the DeepTMHMM (TransMembrane Hidden Markov Model) to predict protein structure^110^.

## Supplementary Information

Supplementary Information.

## Data Availability

The PacBio CLR raw reads for *C. gunnari* and *C. esox* are available from NCBI under BioProject PRJNA857989; *T. borchgrevinki* raw reads are available under BioProject PRJNA907802. The mitogenome assemblies and annotations presented in this study are hosted on Dryad (https://doi.org/10.5061/dryad.9ghx3ffn0j [Temporary reviewer link: https://datadryad.org/stash/share/rtt08g4kjSvu7TBWUK7vkRRX2_bsHToFh1RBynABQyo]).
