# Supplementary material for: Novel mitochondrial genome rearrangements including duplications and extensive heteroplasmy could underlie temperature adaptations in Antarctic notothenioid fishes
Source: Sci Rep. 2023 Apr 28;13:6939. doi: 10.1038/s41598-023-34237-1 (PMC10147917; doi:10.1038/s41598-023-34237-1)
Supplement: Supplementary file 1 — Supplementary Information. [file 41598_2023_34237_MOESM1_ESM.pdf]

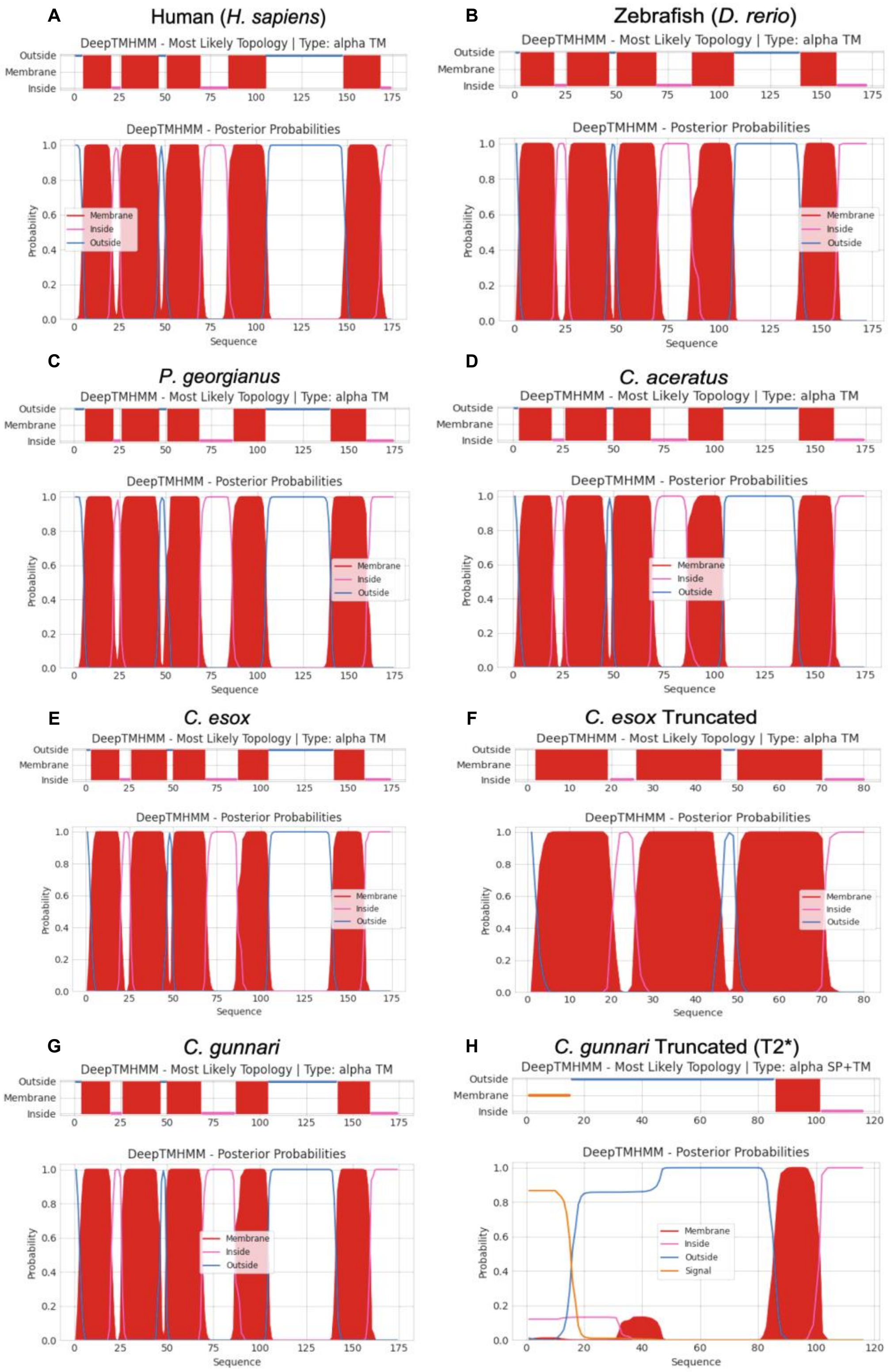

Figure S1. DeepTMHMM transmembrane predictions for ND6 protein in (A) Human, (B) Zebrafish, (C) *P. georgianus*, (D) *C. aceratus,* (E) *C. esox,* (F) *C. esox truncated,* (G) *C. gunnari,* (H) *C. gunnari truncated (T2).*

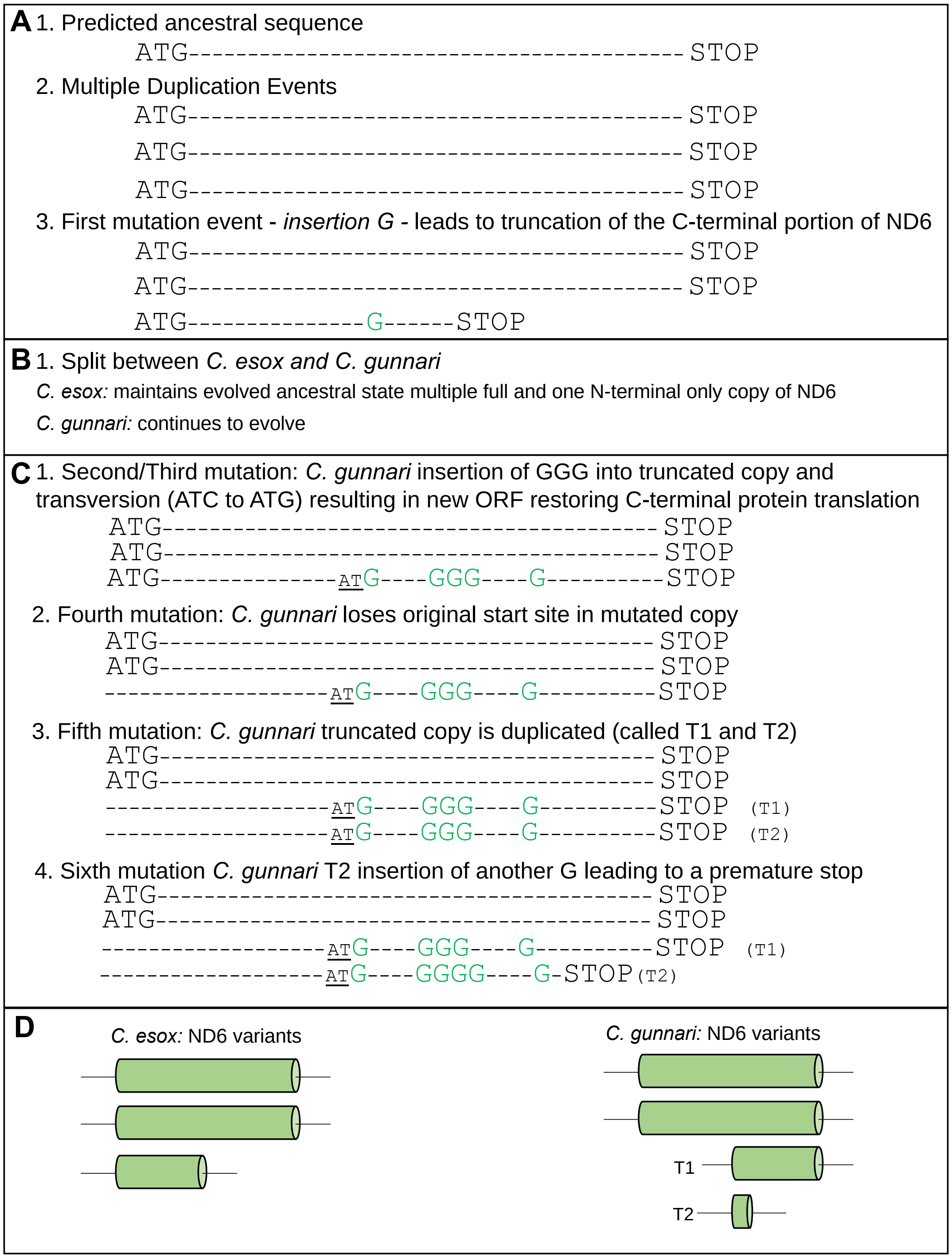

Figure S2. This flow chart represents our step-by-step hypothesis for the most parsimonious explanation of the existence of the various truncated copies of ND6 in *C. esox* and *C. gunnari*. (A) describes events in the common ancestor, (B) describes events at the time of the split between *C. esox* and *C. gunnari* (C) describes evens in *C. gunnari* after the split from *C. esox*. Protein sequences are represented at the nucleotide level beginning with a start codon ATG and ending at STOP. Dashes represent nucleotide stretches of any length. The number of dashes does not correspond to the actual length of the *nd6* sequence. Mutations corresponding to each step are shown in enlarged green font with other unmutated relevant nucleotides shown underlined. (D) describes the possible ND6 proteins present for each species with the truncations aligned to the portion of the full sequence with which they overlap.

Table S1. Repeat information for control region of *C. aceratus*

Indices	Period Size	Copy Number	Consensus Size	Percent Matches	Percent Indels	Score	A	C	G	T	Entropy (0-2)
720--1086	149	2.5	149	98	1	711	28	33	15	21	1.94
720--1086	75	4.9	75	98	1	706	28	33	15	21	1.94

Table S2. Repeat information for control region of *P. georgianus*. (a) CR1, (b) CR2, (c) CR3

(a) CR1

Indices	Period Size	Copy Number	Consensus Size	Percent Matches	Percent Indels	Score	A	C	G	T	Entropy (0-2)
872--1331	52	8.8	53	99	0	858	23	33	6	36	1.81

(b) CR2

Indices	Period Size	Copy Number	Consensus Size	Percent Matches	Percent Indels	Score	A	C	G	T	Entropy (0-2)
872--1281	53	7.8	53	98	0	797	23	33	6	36	1.80

(c) CR3

Indices	Period Size	Copy Number	Consensus Size	Percent Matches	Percent Indels	Score	A	C	G	T	Entropy (0-2)
725--856	63	2.1	63	97	1	248	25	39	6	28	1.80

Table S3. Repeat information for control region of *T. borchgrevinki*

Indices	Period Size	Copy Number	Consensus Size	Percent Matches	Percent Indels	Score	A	C	G	T	Entropy (0-2)
157--448	97	3.0	97	99	0	575	38	14	15	32	1.87
1263--2319	50	21.0	50	77	7	728	36	15	10	37	1.82
1631--2269	152	4.2	152	80	6	783	34	17	11	36	1.87
1779--2361	253	2.3	251	92	1	855	34	17	11	36	1.86
1383--2106	101	7.2	101	95	0	1121	35	16	10	37	1.83
2086--2381	101	2.9	101	97	0	547	33	16	12	36	1.87
1383--2207	202	4.1	199	88	3	872	35	16	10	37	1.83
1383--2207	303	2.7	302	86	2	989	35	16	10	37	1.83
